# *In situ* ligand restraints from quantum-mechanical methods

**DOI:** 10.1107/S2059798323000025

**Published:** 2023-01-20

**Authors:** Dorothee Liebschner, Nigel W. Moriarty, Billy K. Poon, Paul D. Adams

**Affiliations:** a Molecular Biosciences and Integrated Bioimaging Division, Lawrence Berkeley National Laboratory, Berkeley, CA 94720, USA; b Department of Bioengineering, University of California, Berkeley, CA 94720, USA; Institute of Integrative Biology, University of Liverpool, United Kingdom

**Keywords:** macromolecular crystallography, ligand restraints, refinement, quantum mechanics, Quantum Mechanical Restraints

## Abstract

In macromolecular crystallographic structure refinement, ligands present challenges for the generation of geometric restraints due to their large chemical variability, their possible novel nature and their specific interaction with the binding pocket of the protein. Quantum-mechanical approaches are useful for providing accurate ligand geometries, but can be plagued by the number of minima in flexible molecules. In an effort to avoid these issues, the Quantum Mechanical Restraints (QMR) procedure optimizes the ligand geometry *in situ*, thus accounting for the influence of the macromolecule on the local energy minima of the ligand. The optimized ligand geometry is used to generate target values for geometric restraints during the crystallographic refinement. As demonstrated using a sample of >2330 ligand instances in >1700 protein–ligand models, QMR restraints generally result in lower deviations from the target stereochemistry compared with conventionally generated restraints. In particular, the QMR approach provides accurate torsion restraints for ligands and other entities.

## Abstract 2

*In situ* quantum-mechanical optimization of ligand structures provides accurate ligand restraints for macromolecular refinement.

## 1. Introduction

In macromolecular crystallography, the majority of samples diffract to moderate resolution, *i.e.* worse than 2 Å. The consequences of moderate- to low-resolution data are twofold: (i) the Fourier maps lack details of the molecule, so the positions of the atoms cannot be determined precisely, and (ii) the number of reflections is low, leading to low data-to-parameter ratios for refinement. To counteract these challenges, crystallo­graphic refinement programs generally apply geometric restraints: *a priori* knowledge that increases the number of observations, thus improving the ratio of observations to refined parameters. These restraints can be applied to model parameters, such as coordinates (via target geometries), atomic displacement parameters and occupancies, but also to more complex model features, such as secondary-structure elements (Headd *et al.*, 2012 ▸), Ramachandran angles (Oldfield, 2001 ▸; Emsley *et al.*, 2010 ▸; Headd *et al.*, 2012 ▸), the parallelity of atom groups (Sobolev *et al.*, 2015 ▸), noncrystallographic symmetry (NCS; Headd *et al.*, 2014 ▸) and side-chain rotamers. In particular, stereochemical restraints help to keep the model geometry chemically plausible by supplying ideal values for bond lengths, angles, torsions, chirals and planes (Evans, 2007 ▸). For the building blocks of macromolecules, stereochemical restraints are available in libraries, such as the Engh and Huber dictionary (Engh & Huber, 1991 ▸, 2001 ▸) for amino acids, and Gilski *et al.* (2019 ▸) and Clowney *et al.* (1996 ▸) for nucleic acids. It is worth noting that the restraint values in these libraries are still being updated (Malinska *et al.*, 2016 ▸; Moriarty *et al.*, 2016 ▸; Moriarty, Liebschner *et al.*, 2020 ▸).

Aside from their building blocks, macromolecules often contain additional components, such as ligands, covalent modifications, sugars, substrates and solvent molecules. The restraints for common ligands are available in libraries, such as the CCP4 monomer library (Vagin *et al.*, 2004 ▸) or *AceDRG* (Long *et al.*, 2017 ▸), which are used by the *CCP*4 software (Winn *et al.*, 2011 ▸) and the molecular viewer *Coot* (Emsley & Cowtan, 2004 ▸; Emsley *et al.*, 2010 ▸). *Phenix* (Liebschner *et al.*, 2019 ▸) uses a small subset of the CCP4 monomer library as well as its GeoStd library (N. W. Moriarty & P. D. Adams, manuscript in preparation; https://github.com/phenix-project/geostd) for restraints. As uncommon or novel ligands often lack library descriptions, restraints need to be obtained by other means. Dictionary generators such as *eLBOW* (Moriarty *et al.*, 2009 ▸), *Grade* (Smart *et al.*, 2011 ▸) and *AceDRG* (Long *et al.*, 2017 ▸) can programmatically create ligand restraints from various sources. One major source is experimental information from the Cambridge Structural Database (Groom *et al.*, 2016 ▸) or the Crystallography Open Database (Gražulis *et al.*, 2012 ▸) or high-resolution data from the Protein Data Bank (PDB; Berman *et al.*, 2000 ▸). Quantum-mechanical (QM) approaches represent another source for generating chemically accurate geometries (van der Kamp & Mulholland, 2013 ▸; Tzeliou *et al.*, 2022 ▸).

Stereochemical restraints are a computational construct that simplify the description of a chemical structure in refinement. Bond-length and valence-angle restraints have close chemical equivalents, while planarity and torsion restraints are less directly related to chemistry. Nevertheless, planes and torsions are useful to help reproduce the correct stereochemistry. Restraints typically consist of an ideal value and an estimated standard deviation (e.s.d. or sigma) that describe the potential energy surface as a parabola (Evans, 2007 ▸). While this is a simplification (for example, it would be more suitable to describe nonbonded restraints with a Lennard–Jones potential; Lennard-Jones, 1931 ▸), it is a sufficient approximation for the purpose of macromolecular refinement.

While the bond and valence-angle restraints are intuitive, it is worth describing the torsion restraint in more detail. A torsion restraint involves four atoms, typically linearly bonded together (1–2–3–4). The torsion restraint will often favor certain angles around the rotatable bond between atoms 2 and 3. These restraints are useful for representing staggered conformations to minimize the potential energy, such as for the orientation of the H3 propeller in methyl groups. The torsion restraints can also control the overall conformation of a ligand that is composed of several rigid groups with rotational degrees of freedom at the connecting bonds. We note that dihedral angles are usually not strongly restrained because the minimum potential energy *in vacuo* or in solution can change depending on the binding environment in the macromolecule. For example, an H3 propeller may deviate from its ideal staggered conformation to avoid steric hindrance. Therefore, torsion restraints usually have large e.s.d. values, such as 30°, which are much higher than the typical valence-angle e.s.d. of 2°, allowing the torsions to vary significantly from their target value. Furthermore, torsion restraints can be periodic, *i.e.* there can be several, equally plausible target values that account for rotational freedom in *sp* ^2^- and *sp* ^3^-hybridized centers. An example is once again the H3 propeller, where the torsion restraint of an H atom has a periodicity of 3, meaning that there is a potential energy minimum at 60°, 180° (60° + 120°) and 300° (60° + 240°) in the staggered conformation. Unfortunately, torsion restraints and their periodicity have sometimes been inappropriately employed for puckered ring systems. Although for practical purposes ring conformations can often be correctly described by unimodal torsion restraints, in the case of pyranose carbohydrates (Atanasova *et al.*, 2022 ▸) this is not generally the case. To restrain the puckering of rings, *Phenix* uses an alternative approach by adding a list of alternative values for the restraint target (the alternative values being aperiodic as illustrated by the restraints for the amino-acid tryptophan, Trp, in the GeoStd library).

Creating geometry targets is a complex and nuanced task and restraints can display great variability. For example, the idealized coordinates and the standard deviations can vary significantly from one program to the other (see, for example, Fig. 4 in Steiner & Tucker, 2017 ▸). There are several reasons for this geometric diversity. (i) Dictionary generators may interpret the information supplied by the user slightly differently. For example, an aromatic ring can be restrained to be planar via a single planar restraint on all atoms of the ring or via a series of three planar restraints centered on the formal double bonds. (ii) Certain geometric conformations, such as ring puckering and torsion angles between rigid ligand components, depend on the vicinity and binding mode of the molecule, so they cannot be predicted optimally by approaches that do not consider the particular environment. Indeed, when ligand restraints are generated by QM approaches the minimization is typically performed in solvent or in isolation. Unfortunately, this approach cannot predict geometries that depend on the presence of other entities, such as proteins, nucleic acids or metal ions. Therefore, idealized ligands can have dramatically different overall conformations and the ligand geometry may change from the idealized structure upon binding to a macromolecule (Perola & Charifson, 2004 ▸; Hao *et al.*, 2007 ▸).

For the purpose of crystallographic refinement, it is also possible to replace energy gradients from routines based on geometric restraints in libraries with those from physics-based force fields or low-level quantum-mechanics Hamiltonians. For the entire macromolecule, *Phenix* has the option of using the Amber (Case *et al.*, 2018 ▸) and OPLS3e (Roos *et al.*, 2019 ▸) molecular-mechanics force fields (Moriarty, Janowski *et al.*, 2020 ▸; Zundert *et al.*, 2021 ▸). The *Q|R* package makes it possible to derive model geometry restraints for refinement from *ab initio* quantum-chemical calculations (Zheng *et al.*, 2017 ▸, 2020 ▸; Wang *et al.*, 2020 ▸). Another approach is to focus the QM method on the ligand and to use non-QM methods for the rest of the model. This procedure is available in the *Phenix AFITT* and *DivCon* modules, which use the MMFF-94 molecular-mechanics force field (Janowski *et al.*, 2016 ▸) and the PM6 quantum-mechanical Hamiltonian (Borbulevych *et al.*, 2014 ▸), respectively. While procedures for obtaining energy gradients for the entire macromolecular model should give accurate ligand geometries (as they take the binding environment into account), the disadvantage is that the calculations can be computationally expensive. Focusing the QM method on the ligand, such as in the case of *AFITT*, reduces the computing expense but does not include surrounding entities.

Here, we present an approach to generate ligand restraints that is a compromise between QM minimization in either solvent or the full macromolecular model. In the new method, the ligand geometry is optimized in the binding pocket, thus considering the immediate surroundings such as neighboring protein residues and water molecules. This approach, the Quantum Mechanical Restraint (QMR) procedure, significantly reduces computing expense compared with optimizing the entire macromolecule, while also providing better restraints than in the GeoStd library.

## 2. Materials and methods

To test the performance of the QMR restraints compared with standard restraints from GeoStd, we carried out refinements of ligand–protein complexes using each set of restraints. Deviations from ideal geometry were compared for each of the two approaches (refinement with GeoStd versus QMR restraints). As test cases, we used protein models from the PDB that have ligands with large r.m.s.d. values for bonds, angles and/or torsions.

### 2.1. The QMR approach

**Figure 1:**
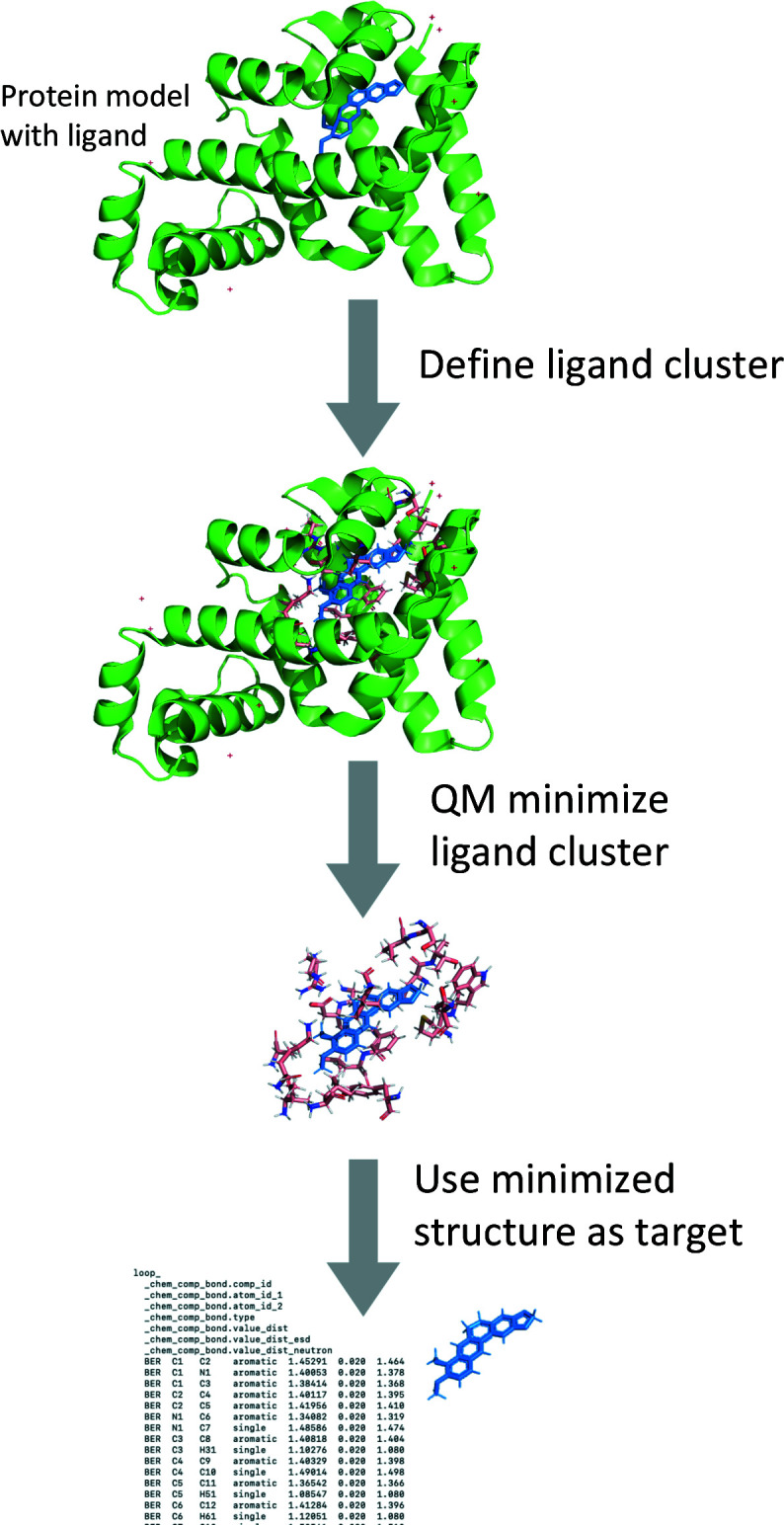
Workflow of the QMR procedure.

The novelty of the QMR approach is to perform QM optimization of the ligand and its immediate environment *in situ*. The first step of the QMR procedure (Fig. 1 ▸) is to define the subset of the macromolecular model whose geometry is to be optimized. This subset is hereafter denoted the ‘ligand cluster’. The ligand cluster is obtained by selecting the ligand as well as all entities, including those related by crystallo­graphic symmetry operations, within a cutoff radius around the ligand. The default radius parameter is 3.5 Å, but can be changed by the user. This radius includes entities within hydrogen-bond or van der Waals interaction distance, but also minimizes the ligand-cluster size so that QM minimization is performed quickly. Entities around the ligand are selected in their entirety. For example, if one atom of a protein residue lies within the cutoff radius, the entire residue is part of the ligand cluster. Being an all-electron method, QM minimization requires the ligand cluster to be correctly protonated, *i.e.* the charge states of the ligand, the macromolecular components and water molecules should be correct and complete. The residues in the ligand cluster and the ligand itself should be protonated as necessary. Extracting the ligand cluster from the protein polymer results in unterminated (dangling) bonds. Therefore, the QMR procedure optionally terminates these with charge-neutral moieties by default or optionally zwitterions. The neutral approach reduces convergence issues and prevents H atoms from moving away from their parents during QM minimization (‘fly-away’ protons). In macromolecular X-ray crystallo­graphy, water molecules are typically modeled as O atoms (instead of complete water molecules), so they are protonated as part of the QMR procedure. Geometry minimization of the ligand cluster is by default performed with the PM7 method of *MOPAC* (Stewart, 1990 ▸, 2016 ▸). Optionally, PM6-D3H4 can be used (Řezáč & Hobza, 2012 ▸). Higher level QM basis sets are available with the third-party open-source QM package *Orca* (Neese, 2012 ▸, 2018 ▸). The ligand geometry obtained after QM minimization is used to generate the target values for the geometric restraints in the standard crystallo­graphic refinement procedure (Afonine *et al.*, 2012 ▸), with e.s.d. values derived from the corresponding GeoStd restraints. The QMR procedure can be performed during a run of *phenix.refine* (Afonine *et al.*, 2012 ▸) or separately as a command-line script. In either mode the results of the QM minimization can be written and read from file, so that the calculations need only be run once. The QMR ligand restraints can also be written to file. We note that the generation of restraints with QMR does *not* use experimental data in any way.

### 2.2. Screening the PDB for examples

We analyzed protein–ligand complexes in the PDB to find suitable test cases for the application of QMR restraints. The screening process started with all protein models that had at least one ligand, without RNA/DNA, with deposited X-ray diffraction data up to at least 3 Å resolution and a molecular weight of less than 2000 kDa. The purpose of these criteria was to speed up the computations and minimize manual interventions. For example, ligands are difficult to place at low resolution (worse than 3 Å) and they may need to be validated manually. As the termination of nucleic polymers is generally unknown and the protonation is thus undetermined, the QM minimization is challenging for RNA/DNA. Therefore, we focused on protein-only entries.

We then refined the set of test cases by focusing on models that are suitable for the application of the QMR method. We used the following criteria, principally to satisfy the need for a complete ligand cluster to make the quantum-mechanical minimization feasible.

(i) The ability of *Phenix* tools to process the model/data without manual intervention.
(ii) Properties of the cluster. No other ligands, metals or alternate conformations within 5.5 Å of the ligand of interest prior to H-atom addition. No missing atoms within 3.5 Å of the ligand after H-atom addition. This filtering was performed to simplify the interpretation of the results. Validating ligands, metals and alternative conformations can be complex and may require manual inspection, which is beyond the scope of this work, so we analyzed ligands in well defined environments.
(iii) Properties of the ligand: map–model correlation of at least 0.7, number of non-H atoms between eight and 40, a total charge of −1, 0 or +1 and a mean r.m.s.d. of at least 0.2 Å for bonds, 5° for angle or 30° for torsion angles after performing benchmark refinements with standard restraints (see Section 2.3). A cutoff value of 0.7 for the map–model correlation is a very simple ligand validation measure, as a lower correlation coefficient may be indicative of an incorrectly placed ligand. Similarly, we investigated models with a charge of −1, 0 or +1, as large charges may suggest incorrect protonation. Ligand geometries with large deviations suggest that the GeoStd restraints were inadequate in some way, which makes them suitable test cases for the QMR method. Finally, we ignored common crystallization-solution components, such as 2-(*N*-morpholino)ethanesulfonic acid (MES) and polyethylene glycol (PEG) molecules.

The filtering resulted in a set of test cases containing 2334 ligands in 1712 models. These models were refined with standard restraints (Section 2.3) and with QMR restraints (Section 2.4) to compare their performance.

### 2.3. Benchmark refinements

Before applying the QMR approach, each model was first refined against the deposited diffraction data with *phenix.refine* using standard restraints. We used the current best *Phenix*-based ligand restraints, *i.e.* QM-calculated restraints validated by *Mogul*, as available in the GeoStd database (N. W. Moriarty & P. D. Adams, manuscript in preparation; https://github.com/phenix-project/geostd). *Mogul* (Bruno *et al.*, 2004 ▸; Cottrell *et al.*, 2012 ▸) is a program for molecular geometry based on the Cambridge Structural Database (CSD; Groom *et al.*, 2016 ▸) which provides preferred values of bond lengths, valence angles and torsion angles and can be used to either derive restraints for small molecules or to validate their geometry. By performing these benchmark refinements, changes in the ligand geometry after refinement with QMR restraints (Section 2.4) are solely caused by the QMR method. The refinement strategy was as follows. The protein–ligand complexes were refined with ten macrocycles of *phenix.refine*. Nondefault parameters were geometry weight optimization and atomic displacement parameter (ADP) refinement according to the resolution of the diffraction data. For resolutions worse than 1.5 Å, all atoms were refined with isotropic ADPs. For resolutions between 1.5 and 1.2 Å, protein atoms were refined as anisotropic and other ‘heavy’ atoms (such as water) were refined as isotropic. For resolutions better than 1.2 Å, all non-H atoms were refined with anisotropic ADPs. We then extracted the minimum, maximum and mean r.m.s.d. values of bond lengths, angles and torsions for each ligand.

### 2.4. QMR refinements

After benchmark refinements, we refined the models again, this time using QMR restraints for the ligand under investigation instead of the GeoStd restraints. Otherwise, the strategy was the same. The QMR restraints were obtained before the first macrocycle of refinement with *phenix.refine*. As for the benchmark refinements, we extracted the minimum, maximum and mean r.m.s.d. values of bond lengths, angles and torsions for each ligand.

### 2.5. Availability

The QMR procedure can be performed during a run of *phenix.refine* in the GUI or via the command line using a .phil file that can be obtained via a command-line script. QM minimization of the cluster can also be performed separately as a command-line script. Availability of QMR starts with *Phenix* version dev-4753.

## 3. Results and discussion

The following section presents the performance of GeoStd restraints *versus* QMR restraints. Firstly, we describe the search for suitable examples. This is followed by an overall comparison of valence-angle and torsion-angle r.m.s.d. values for ligands using either GeoStd or QMR restraints. Finally, we discuss two examples for ligands where the QMR restraints yield better target values than those in the GeoStd restraints.

Depending on the type of restraints (GeoStd or QMR), deviations from target values after refinement can have different causes. For GeoStd restraints, deviations can be due to the following.

(i) The ligand is strained in the binding environment compared with the local minimum of the isolated ligand used to generate the restraints.
(ii) The information from the experimental data conflicts with how the ligand is modeled. There can be different degrees of this scenario. For example, if parts of the ligand are dis­ordered the r.m.s.d. values may increase (but not necessarily). A more extreme case would be that the ligand is not present in the crystal.
(iii) The restraints are inconsistent with themselves or with the stereochemistry of the ligand.

For QMR restraints, scenario (i) is slightly different. Ligand strain should be addressed by the QM minimization that includes the environment. Therefore, if there are still large deviations using QMR restraints, it could mean that the environment used for the QM minimization was incomplete. For example, it may be that a water molecule was missing, a side chain was modeled incorrectly or the ligand is exposed to disordered bulk solvent (rather than explicitly modeled water molecules).

### 3.1. Comparing QMR and GeoStd restraints

To compare the performance of GeoStd restraints versus QMR restraints, we analyzed the bond-length, valence-angle and torsion-angle r.m.s.d. values of the ligands after refinement with each set of restraints. We note that the ideal values of the GeoStd and QMR restraints are generally different. Therefore, two identical geometries can produce different r.m.s.d. values because the r.m.s.d. for each set of restraints is calculated against the corresponding ideal values.

**Figure 2:**
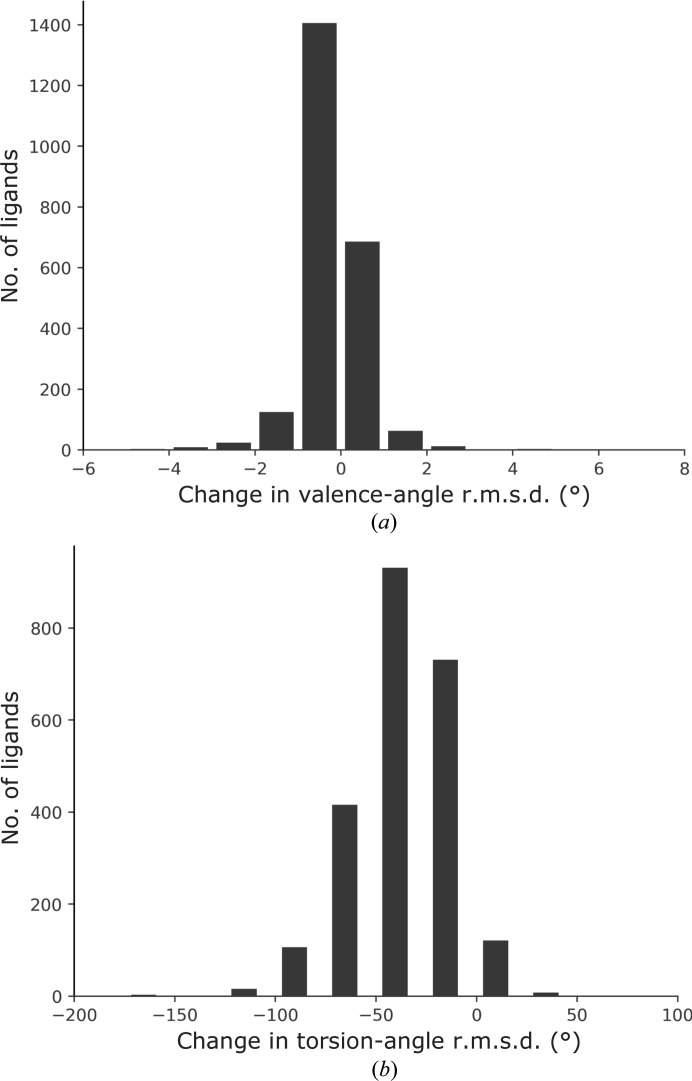
Changes in valence-angle r.m.s.d. (*a*) and torsion-angle r.m.s.d. (*b*).

Fig. 2 ▸ shows the changes in valence-angle r.m.s.d. (Fig. 2 ▸ *a*) and torsion-angle r.m.s.d. (Fig. 2 ▸ *b*). For valence angles, the r.m.s.d. improved by up to 2° in more than 1500 ligands. For torsion angles the improvement is even more pronounced, as the r.m.s.d. decreased for the majority of ligands, by up to 180°, while only a small fraction displayed a deterioration. In contrast to valence and torsion angles, bond lengths are not expected to change much between GeoStd and QMR restraints (Supplementary Fig. S1). Indeed, the changes in r.m.s.d. are mostly within 0.02 Å and are almost equally distributed around zero. The usage of QMR restraints therefore leads to lower valence-angle and torsion-angle r.m.s.d. values overall.

**Figure 3:**
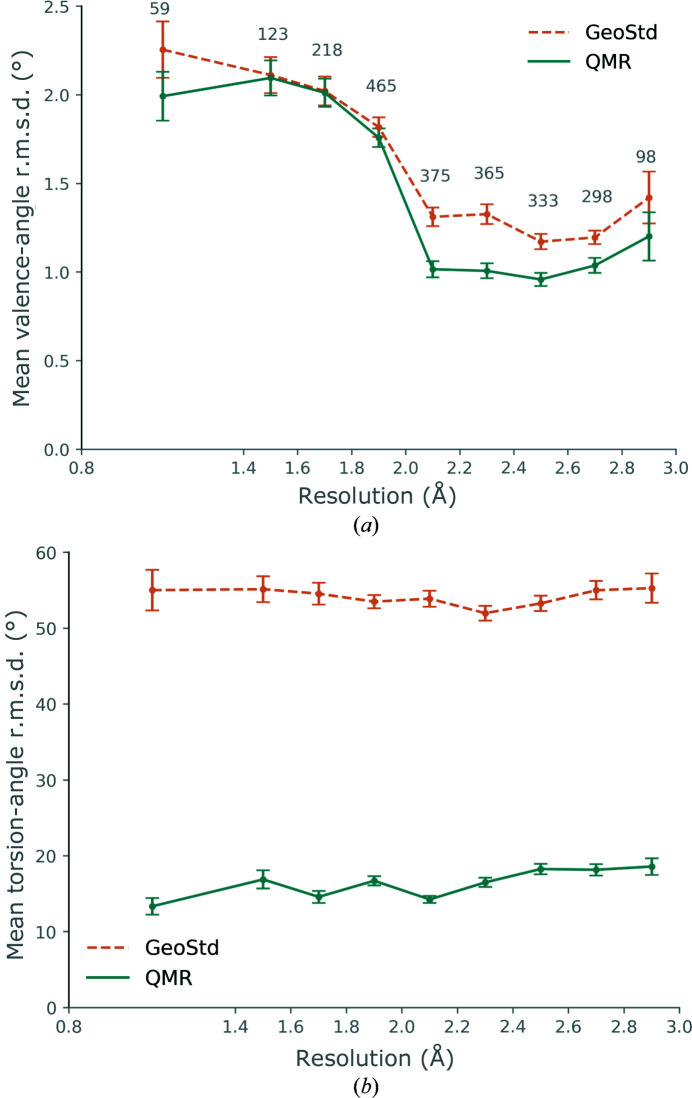
Mean valence-angle r.m.s.d. (*a*) and mean torsion-angle r.m.s.d. (*b*) for ligands averaged in resolution bins. The highest resolution bin is 0.8–1.4 Å; the other bins have a 0.2 Å width. The number of ligands per bin is indicated above the orange line in (*a*). The error bars represent the standard error of the mean.

As the weight on geometric restraints depends on the data resolution in refinement, we also analyzed the resolution dependence of the ligand r.m.s.d. values. Fig. 3 ▸ shows the mean valence-angle (Fig. 3 ▸ *a*) and torsion-angle (Fig. 3 ▸ *b*) r.m.s.d. averaged in resolution bins. The mean bond-length r.m.s.d. averaged by resolution bin is shown in Supplementary Fig. S2. The plots include the standard error of the mean (s.e.m.) represented by error bars. The s.e.m. represents the accuracy of the mean. For the mean r.m.s.d. values for valence angles, we can distinguish three regions in the plot, according to the closeness of the mean of the distributions: the high-resolution bin covering better than 1.4 Å, the medium-resolution bins covering 1.4–2 Å and the medium- to low-resolution bins covering 2–3 Å. We note that the s.e.m. regions overlap for the highest (0.8–1.4 Å) and the lowest (2.8–3 Å) resolution bins. We therefore also performed a *t*-test and computed *p*-values to find out whether there is a difference between the means of the two distributions (Supplementary Table S1).

For the high-resolution bin, the mean r.m.s.d. values differ by ∼0.3 Å with a slight overlap of the s.e.m. regions. The *p*-value is 0.215, indicating that the mean is similar. At high resolution, the weight on geometric restraints is usually less tight, meaning that the experimental data contain sufficient information to drive the model towards a chemically meaningful geometry in its environment (*i.e.* the binding pocket in the protein). It follows that the r.m.s.d. deviations from the restraints are therefore generally greater at high resolution. The curves for both GeoStd and QMR restraints display a sudden drop at 2 Å resolution. Near this resolution, the weight-optimization procedure of *phenix.refine* loosens the weight on geometry restraints somewhat so that the conformations can deviate from ideal targets when justified by the data. The mean r.m.s.d. values for the medium-resolution range agree for the two types of restraints. Therefore, there appears to be little difference between the GeoStd restraints and QMR restraints for valence angles at medium resolution. In contrast, in the medium- to low-resolution range the mean valence-angle r.m.s.d. is systematically lower for QMR restraints than for GeoStd restraints. This is corroborated by the *p*-values, which indicate that there is a difference between the means. An exception is the 2.8–3 Å resolution bin, for which the s.e.m. overlaps, with a *p*-value of 0.274. We note that refining and modeling ligands at such low resolution can be challenging and that the number of ligands in this resolution range is small (98) compared with the other bins.

As discussed in the introduction to this section, there can be different causes for the deviations of refined geometry from the ideal values, which can be difficult to untangle. This is especially true for GeoStd restraints, which cannot distinguish between the scenarios where deviations are large because of ligand strain or inadequate restraints. For QMR restraints, unexplained ligand strain is greatly reduced and thus can generally be excluded as a cause of the deviations. Instead, the deviations can result from incomplete ligand environments, disorder or the nonbonded repulsion terms used in refinement, which are much simpler than the electron calculations used in the QM *in situ* geometry minimization. This is useful information that can help guide further model building. Thus even if QMR restraints do not immediately result in lower r.m.s.d. values, they can help to identify parts of the model that might be improved.

For torsion angles, the mean r.m.s.d. for GeoStd restraints is 50–60° in all resolution bins, while it is 10–20° for QMR restraints. Using QMR restraints therefore leads to a systematic improvement in torsion-angle r.m.s.d. values across all resolution ranges. It is not surprising that the QMR restraints have such a significant effect on the deviations from target values. As discussed in Section 1, torsion angles are the geometric feature that is most difficult to predict. For example, the torsions between rigid building blocks of ligands typically do not have one single energy minimum *in vacuo* or solution. Furthermore, the potential energy surfaces can be broad with low energy transition barriers. Similarly, the conformation of ring puckers cannot be predicted without considering the *in situ* environment. For instance, Atanasova *et al.* (2022 ▸) showed that torsion-angle restraints improve the refinement of carbohydrate residues. We note that the map–model correlation for the ligands is effectively identical before and after refinement (Supplementary Figs. S3 and S4).

### 3.2. Example 1: BER in PDB entry 3vw2

**Figure 4:**
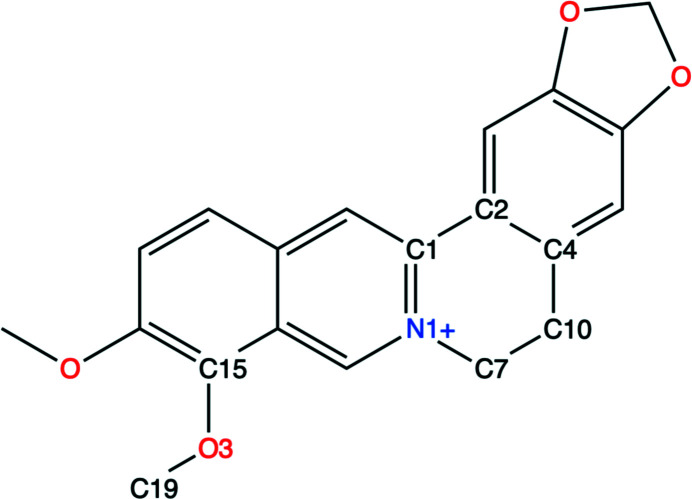
The chemical structure of berberine. Relevant atoms are annotated with PDB atom names.

**Figure 5:**
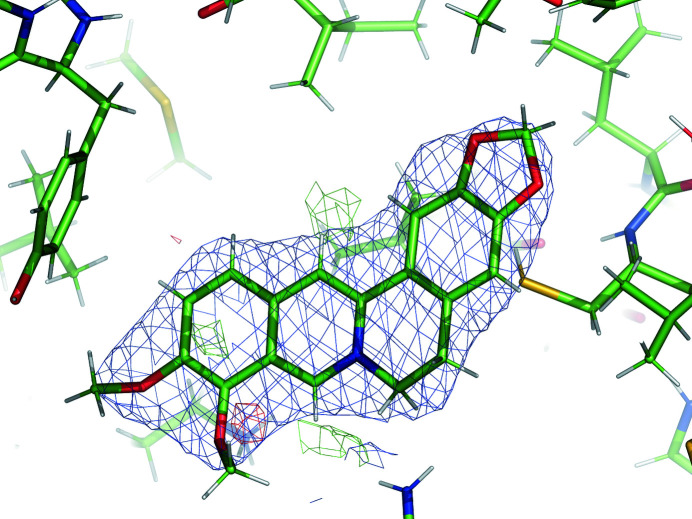
BER *D* in PDB entry 3vw2 and electron density after benchmark refinement with GeoStd restraints. Blue, 2*mF*_obs_ − *DF*_model_ map at 1 r.m.s. and 1.5 Å carve; green/red, *mF*_obs_ − *DF*_model_ map at ±3 r.m.s. and 3 Å carve.

PDB entry 3vw2 is a protein of the TetR family of transcriptional repressors (Yamasaki *et al.*, 2013 ▸) which can regulate the expression of multidrug transporter genes. The dimeric structure contains one copy of the ligand berberine (BER) in each chain. In this example, we discuss the berberine molecule associated with chain *D*. The chemical structure of BER is shown in Fig. 4 ▸. Berberine is a heteropentacyclic compound with two methoxy groups connected to a phenyl ring. The phenyl rings are expected to be flat, while the unsaturated six-membered ring may be puckered. The CH_3_ ends of the methoxy groups have a rotational degree of freedom. The resolution of the diffraction data is 2.34 Å. The medium-resolution density supports the placement of the BER molecules (Fig. 5 ▸), with a map–model correlation coefficient of 0.76 for BER *D* for the deposited model.

Table 1 ▸ lists the bond, angle and dihedral r.m.s.d. values, targets and deviations for the most significant outliers when using GeoStd restraints compared with QMR restraints in refinement. Table 2 ▸ lists the r.m.s.d. values computed over all available restraints in the ligand. There are no noteworthy bond-length deviations in the models for either set of restraints. The bond-length r.m.s.d. is 0.007 Å for GeoStd restraints; after QMR refinement, the bond-length r.m.s.d. improved to 0.003 Å. For individual bonds, there are no large deviations. For GeoStd restraints, the C20—O4 and C19—O3 bonds deviate by 0.025 and 0.021 Å, respectively. The C20—O4 bond has also the largest deviation for QMR restraints, but it decreased to 0.015 Å.

The valence-angle r.m.s.d. values show significant improvement. The angle r.m.s.d. for all non-H angles decreases from 2.4° with GeoStd restraints to 0.8° with QMR restraints. In the structure from GeoStd refinements, three angles display deviations larger than 5° from ideal: C10—C7—N1 (7.6°), C4—C10—C7 (6.7°) and C15—O3—C19 (6.1°). The first two angles are in the six-membered ring with nitrogen; the third angle is in a methoxy group. The latter is clearly due to the protein environment influencing the conformation. For all three angles, the deviations decrease to less than 2.5° when the model is refined with QMR restraints. We note that the QMR approach modifies the targets of these angles by up to 2.6° compared with the GeoStd restraints (angle C15—O3—C19). We also note that the refined values of the angles differ by several degrees between the two models.

**Figure 6:**
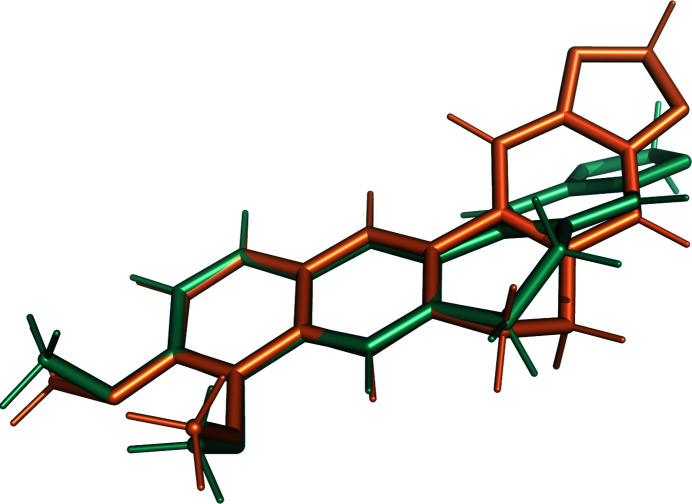
Superposition of the computed structures for ligand BER used for restraint generation in the GeoStd (orange) and QMR (teal) methods.

**Figure 7:**
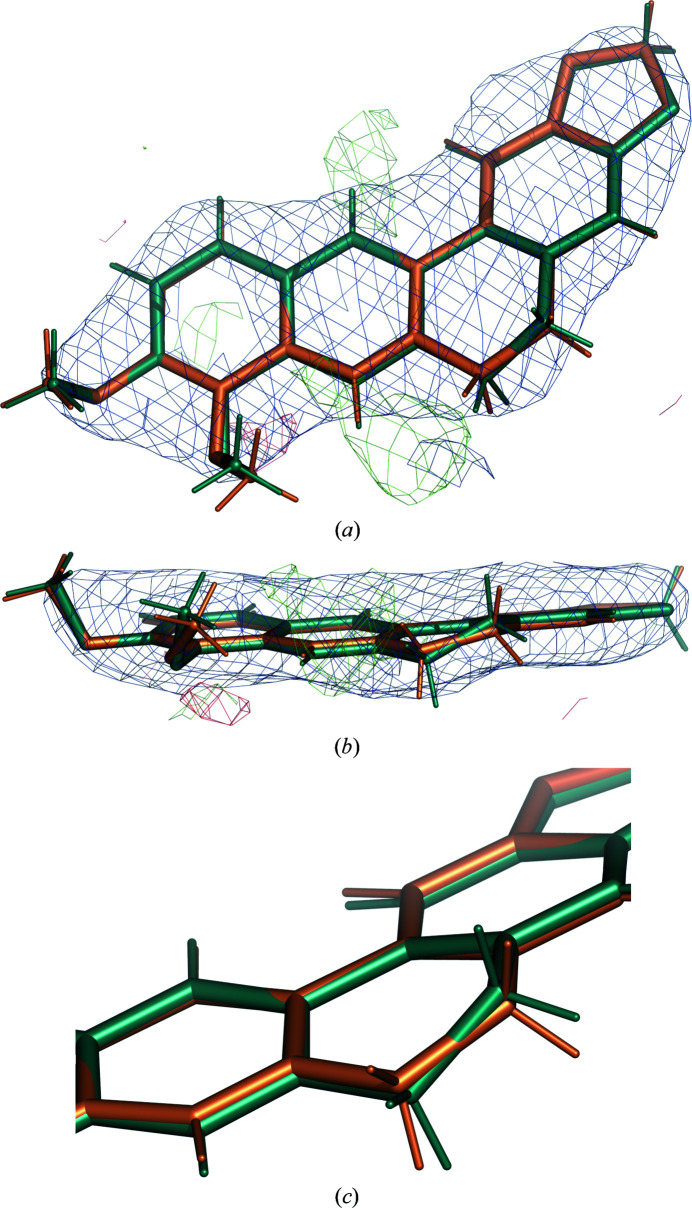
(*a,* *b*) BER *D* in PDB entry 3vw2 after refinement with QMR restraints (teal) and after refinement with GeoStd restraints (orange). The electron density is computed with the model from QMR refinement. Blue, 2*mF*_obs_ − *DF*_model_ map at 1 r.m.s. and 1.5 Å carve; green/red, *mF*_obs_ − *DF*_model_ map at ±3 r.m.s. and 2 Å carve. (*c*) Close-up of the central unsaturated six-membered ring.

The dihedral angles show the largest improvement. The r.m.s.d. for all non-H torsion restraints decreases from 48.1° with GeoStd restraints to 7.1° with QMR restraints. For GeoStd restraints, three dihedral angles display deviations larger than 30° from ideal: the deviations for C4—C10—C7—N1, C1—C7—N1—C10 and C2—C10—C4—C7 are 76.7°, 56.1° and 48.2°, respectively. All three torsion restraints describe the central unsaturated six-membered ring. We note that the two largest GeoStd bond-angle deviations were also in this ring. After refinement with QMR restraints, the dihedral deviations decrease to 10.4°, 1.0° and 11.4°, respectively. Fig. 6 ▸ shows a superposition of the idealized structures from GeoStd and from QMR. While the overall structure is very similar, the ideal geometries differ in the puckering of the unsaturated six-membered ring. The electron density clearly favors the puckering from the QMR restraints and refinement with both sets of restraints thus resulted in similar structures (Figs. 7 ▸ *a* and 7 ▸ *b*). The largest deviation occurs in the unsaturated six-membered ring (Figs. 7 ▸ *b* and 7 ▸ *c*), which is most likely to be a result of the different puckers in the idealized structures. For GeoStd restraints, the electron density drove refinement away from the ideal target structure, resulting in large bond-angle and torsion-angle deviations. In contrast, the QMR restraints predicted the correct pucker and thus yielded significantly lower deviations from the ideal target values. Therefore, QMR restraints provide more adequate restraints for the BER molecule than GeoStd restraints because they can predict the correct pucker for the unsaturated six-membered ring.

### 3.3. Example 2: EYW in PDB entry 6gh7

**Figure 8:**
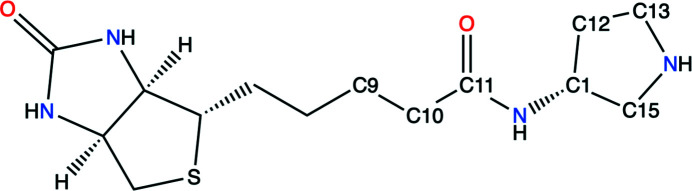
The chemical structure of EYW {5-[(3*aS*,4*S*,6*R*)-2-oxidanylidene-1,3,3*a*,4,6,6*a*-hexahydrothieno[3,4-*d*]imidazol-4-yl]-*N*-[(3*R*)-pyrrolidin-3-yl]pentanamide}. Relevant atoms are annotated with PDB atom names.

**Figure 9:**
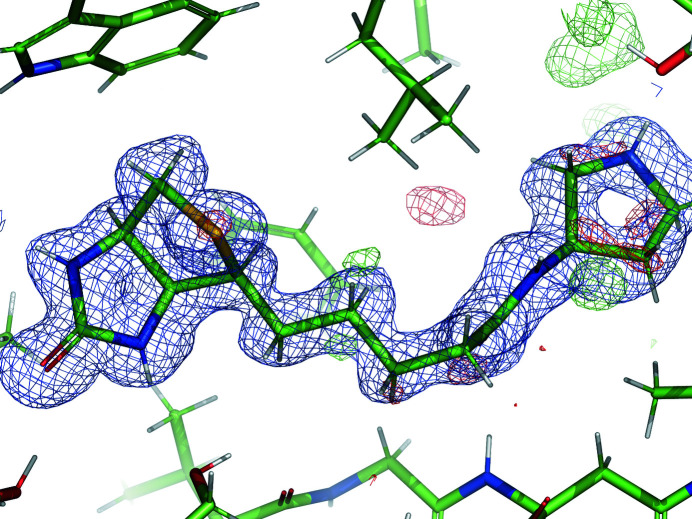
EYW of chain *A* in PDB entry 6gh7 and electron density after refinements with GeoStd restraints. Blue, 2*mF*_obs_ − *DF*_model_ map at 1 r.m.s.; green/red, *mF*_obs_ − *DF*_model_ map at ±3 r.m.s.

PDB entry 6gh7 is a complex of streptavidin with a biotinylated pyrrolidine (Nödling *et al.*, 2018 ▸). The homotetrameric structure contains one copy of the ligand EYW in each chain. In this example, we discuss the EYW molecule associated with chain *A*. The chemical structure of EYW, shown in Fig. 8 ▸, contains a pyrrolidine ring at one end with a fused imidazole and a tetrahydrothiophene ring with a common C—C bond at the other end. The resolution of the diffraction data is 1.08 Å and the Fourier maps show clear peaks for each atom of the EYW molecule (Fig. 9 ▸), making the placement of the ligand unambiguous. Before benchmark refinement, the map–model correlation coefficient was 0.86.

Table 3 ▸ lists the bond, angle and dihedral r.m.s.d. values, targets and deviations for the most significant outliers when using GeoStd compared with QMR restraints (the r.m.s.d. values are shown in Table 2 ▸). The bond-length deviations are not noteworthy for either set of restraints. For GeoStd restraints, only one bond has a deviation greater or equal to 0.020 Å, which is C1—C15 (with a deviation of 0.020 Å). This bond deviation increased slightly to 0.031 Å for QMR restraints. Furthermore, the C1—C12 bond deviation increased from 0.018 Å for GeoStd restraints to 0.028 Å with QMR restraints.

The valence angles remained quite similar for both sets of restraints. The angle r.m.s.d. for all non-H angles increased slightly from 2.0° with GeoStd restraints to 2.2° with QMR restraints. The largest valence-angle outlier occurs for the C15—C1—N12 angle, which is 7.2° larger than the target value. This angle is between the pyrrolidine ring and the neighboring peptide group. For QMR restraints, the deviation for the C15—C1—N12 angle decreased to 5.0°.

**Figure 10:**
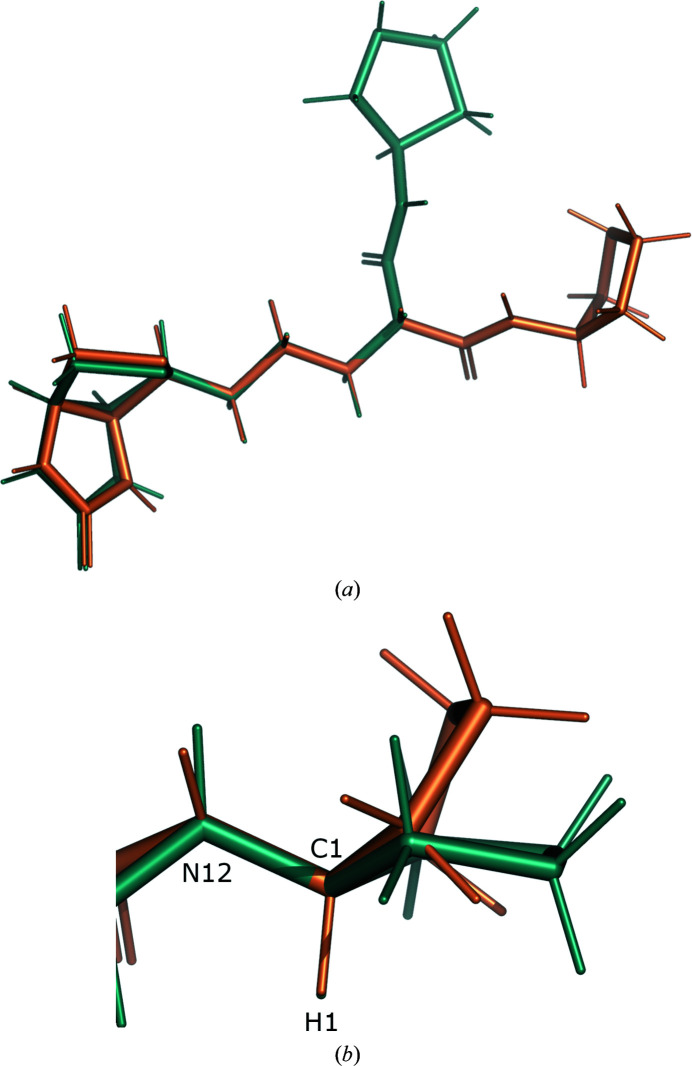
Superposition of the idealized EYW structures from GeoStd (orange) and QMR (teal) restraints. (*a*) Entire molecule; superposition based on the alkane group. (*b*) Close-up of the pyrrolidine ring; superposition based on the N12, C1 and H1 atoms.

The most significant changes occurred for the torsion restraints. The torsion-angle r.m.s.d. for all non-H angles decreases from 33.7° with GeoStd restraints to 4.5° with QMR restraints. Three dihedral angles have deviations larger than 30° for GeoStd restraints: C9—C10—C11—N12 (67.6°), C1—C15—N14—C13 (53.6°) and C1—C12—C13—N14 (38.4°). The C9—C10—C11—N12 dihedral describes the rotation between the peptide unit and the alkane group. The other two dihedral angles describe the pucker of the pyrrolidine ring. The deviations of all three torsion angles decrease to less than 3.5° after refinement with QMR restraints. While the refined values of the dihedral angles are very similar (within 1°), the ideal values of the QMR restraints are different. Fig. 10 ▸ shows superposition of the idealized GeoStd structure and the QM-minimized structure. The rotamer of the C9—C10—C11—N12 torsion angle is very different for GeoStd and QMR restraints (Fig. 10 ▸ *a*). Furthermore, the QM minimization predicts the pyrrolidine ring to have a pucker facing ‘down’, while the GeoStd restraints have the pucker facing ‘up’ (Fig. 10 ▸ *b*).

**Figure 11:**
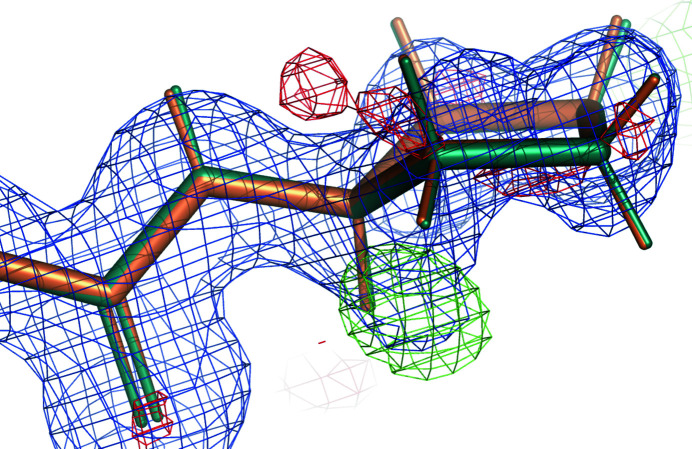
EYW *A* in PDB entry 6gh7 after refinement with QMR restraints (teal) and after refinement with GeoStd restraints (orange). The electron density is computed with the model from QMR refinement. Blue, 2*mF*_obs_ − *DF*_model_ map at 1 r.m.s. and 1.2 Å carve; green/red, *mF*_obs_ − *DF*_model_ map at ±3 r.m.s. and 3 Å carve.

The refined structures of EYW with GeoStd and QMR restraints are basically identical (Fig. 11 ▸). Indeed, as the resolution of the diffraction data is very high (1.08 Å), the weight on the GeoStd restraints is less tight and the experimental data could drive the model towards a chemically meaningful geometry in its environment. We note that the positive difference density peak close to the H1 atom of the pyrrolidine ring as well as the smaller negative peaks in the same area (Fig. 11 ▸) suggest some disorder or alternative conformations of the pyrrolidine ring. This part of the ligand is exposed to water molecules and bulk solvent, so it may be prone to disorder. That may also explain why the bond and angle deviations increased slightly for QMR restraints. As the QMR minimization only accounts for explicitly modeled water molecules, the geometry of EYW may have relaxed into the bulk-solvent area. Nevertheless, the QMR restraints provide overall better targets for the conformation of the EYW molecule, *i.e.* the orientation of the rigid groups with respect to each other and the puckering of the pyrrolidine ring.

### 3.4. Practical considerations

QMR restraints can be generated at any stage of the refinement process once the binding pocket has been well defined and all surrounding atoms are present in the model. There are two reasons for the necessity of completeness. The first is fundamental to quantum methods. QM is an all-electron method that calculates molecular orbitals (and thereafter molecular geometries) based on the electrons present. Any missing electrons result in a very different calculated solution; ergo the geometry is not that found in the crystal. The second is related to the QMR algorithm. The ligand is free to move in the protein environment. If the binding pocket is ill-formed, the ligand may move to a less ideal position in the model. It is therefore prudent to inspect the final geometry of the ligand cluster.

However, as refinement progresses, the macromolecular/solvent environment of the ligand may change, perhaps even significantly. In such cases, it is advisable to regenerate the QMR restraints. There is also a parameter to calculate the QMR restraints for each macrocycle of the refinement. One QMR option allows the protein side chains to optimize along with the ligand to accommodate larger pocket movements and initial ligand geometries in a tight space. The typical running time of the QMR minimization is approximately 15 min on an M1 MacBookPro.

The QMR procedure may be helpful for non-expert users who are dealing with flexible ligands and who are not familiar with the options in ligand restraint-generator programs that can tweak the ligand geometry towards a conformation that fits into the binding environment.

## 4. Conclusion

We present a new method to generate ligand restraints that makes use of *in situ* QM minimization of the ligand within its binding environment. This procedure can be applied to ligands as well as covalently bound entities including carbohydrates and other post-translational modifications. Refinement of 1712 models shows that QMR-restrained parameters generally have lower deviations from their ideal values compared with using conventionally generated restraints from the GeoStd library. Valence-angle restraints are generally improved, whilst torsion-angle restraints are significantly better. Two examples (BER in PDB entry 3vw2 and EYW in PDB entry 6gh7) illustrate how QMR restraints provide a target ligand geometry that fits better to the protein environment than the GeoStd restraints at both medium and high resolution.

One feature of the QMR procedure that is beyond the scope of this article is the calculation of energies. To quantify the reduction in strain of the ligand geometry, the strain energy can be calculated pre- and post-refinement. Furthermore, the energy values of the entire cluster can be used to quantify its improvement in geometry. Comparing the energies of different ligand poses in the cluster may help to determine the most likely ligand orientation. A particular application of these comparative energies is in the analysis of rotamers, for example histidine protonation states and orientations. This is currently under investigation.

## Supplementary Material

Supplementary Figures and Table. DOI: 10.1107/S2059798323000025/ai5010sup1.pdf

## Figures and Tables

**Table 1:** Bond lengths, valence angles and torsion angles for BER in PDB entry 3vw2 with more than 0.02 Å, 5° and 30° deviation from ideal, respectively If the values are above the cutoff for at least one set of restraints (GeoStd or QMR), they are listed for both refinements to allow comparison. Bond values are in Å, while bond angles and torsions are in degrees.

		GeoStd			QMR		
	Large deviations	Target	Actual	Δ	Target	Actual	Δ
Bonds	C20—O4	1.419	1.394	0.025	1.434	1.419	0.015
	C19—O3	1.430	1.409	0.021	1.437	1.440	−0.003
Angles	C10—C7—N1	109.7	117.3	−7.6	111.7	113.9	−2.2
	C4—C10—C7	109.0	115.7	−6.7	110.9	113.2	−2.4
	C15—O3—C19	115.2	121.3	−6.1	112.6	113.9	−1.3
Torsions	C4—C10—C7—N1	58.0	−18.7	76.7	−50.7	−40.3	−10.4
	C1—C7—N1—C10	43.0	−13.1	56.1	−26.9	−27.9	1.0
	C2—C10—C4—C7	35.8	−12.4	48.2	−39.0	−27.7	11.4

**Table 2:** Bond-length, valence-angle and torsion-angle r.m.s.d. for BER in PDB entry 3vw2 and for EYW in PDB entry 6gh7 Bond values are in Å, while bond angles and torsions are in degrees.

	GeoStd			QMR		
	Bonds	Angles	Torsions	Bonds	Angles	Torsions
BER	0.007	2.4	48.2	0.003	0.8	7.1
EYW	0.010	2.0	33.7	0.013	2.2	4.5

**Table 3:** Bond lengths, valence angles and torsion angles for EYW in PDB entry 6gh7 with more than 0.02 Å, 5° and 30° deviation from ideal, respectively If the values are above the cutoff for at least one set of restraints (GeoStd or QMR), they are listed for both refinements to allow comparison. Bond values are in Å, while bond angles and torsions are in degrees.

		GeoStd			QMR		
	Large deviations	Target	Actual	Δ	Target	Actual	Δ
Bonds	C1—C15	1.534	1.514	0.020	1.551	1.521	0.031
	C1—C12	1.530	1.548	−0.018	1.546	1.574	−0.028
Angles	C15—C1—N12	111.6	118.8	−7.2	112.9	117.9	−5.0
Torsions	C9—C10—C11—N12	210.9	143.3	67.6	142.6	144.2	−1.6
	C1—C15—N14—C13	37.2	−16.4	53.6	−13.8	−16.2	2.4
	C1—C12—C13—N14	−6.5	31.8	−38.4	27.0	30.4	−3.4
