# Supplementary material for: In situ ligand restraints from quantum-mechanical methods
Source: Acta Crystallogr D Struct Biol. 2023 Jan 20;79(Pt 2):100–10. doi: 10.1107/S2059798323000025 (PMC9912925; doi:10.1107/S2059798323000025)
Supplement: Supplementary file 1 [file d-79-00100-sup1.pdf]

STRUCTURAL BIOLOGY

Volume 79 (2023)

Supporting information for article:

In situ ligand restraints from quantum-mechanical methods

Dorothee Liebschner, Nigel W. Moriarty, Billy K. Poon and Paul D. Adams

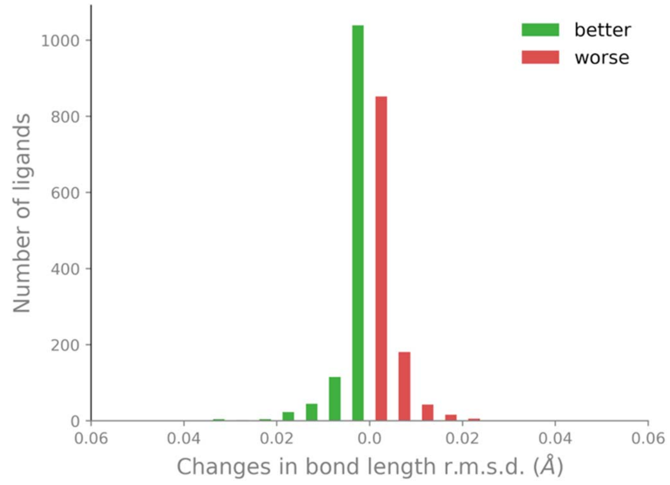

Figure S1 Changes in bond length r.m.s.d. The green bars represent improvement, i.e., the angle r.m.s.d. after refinement with QMR restraints decreased. The red bars represent deterioration, i.e., the bond r.m.s.d. Increased.

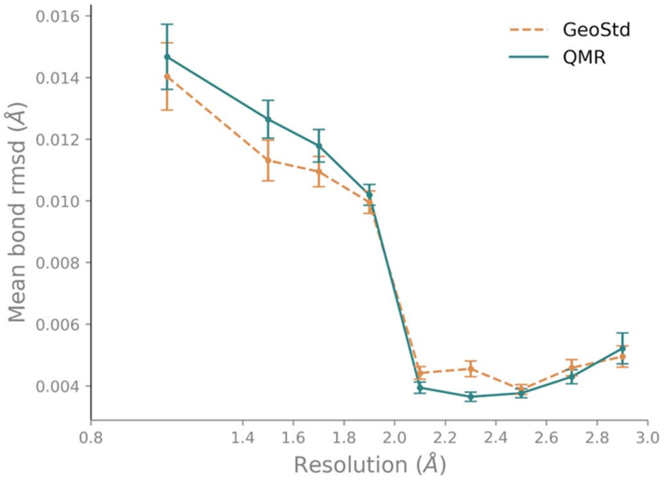

Figure S2 Mean bond length r.m.s.d. for ligands, averaged in resolution bins for GeoStd restraints (orange) and QMR restraints (teal). The highest resolution bin is 0.8Å-1.4Å, the other bins have a 0.2Å width. The error bars represent the s.e.m.

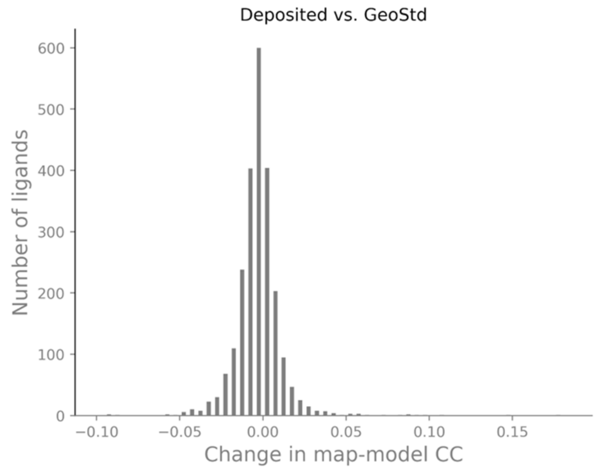

Figure S3 Change in ligand map correlation before refinement (deposited model) and after refinement with GeoStd restraints.

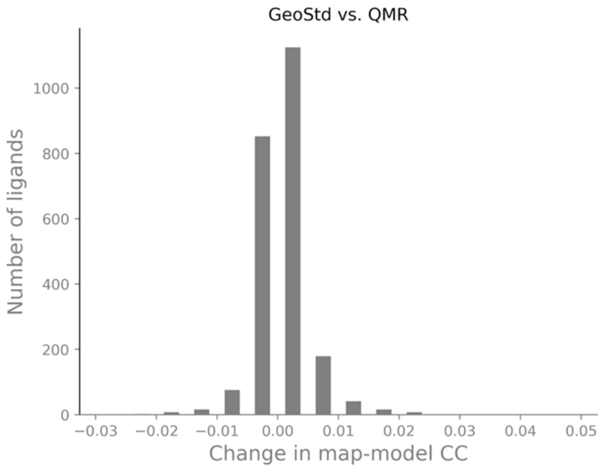

Figure S4 Change in ligand map correlation after refinement with GeoStd restraints and after refinement with QMR restraints.

Table S1 p-value for the mean valence angle r.m.s.d. for ligands, averaged in resolution bins for GeoStd restraints and QMR restraints.

**bin limits (Å)**	**number of ligands**	**p-value**
0.8-1.4	59	0.215
1.4-1.6	123	0.909
1.6-1.8	218	0.930
1.8-2.0	465	0.426
2.0-2.2	375	2.27E-05
2.2-2.4	365	6.23E-06
2.4-2.6	333	1.67E-04
2.6-2.8	298	0.006
2.8-3.0	98	0.274
